# Linkage between Fitness of Yeast Cells and Adenylate Kinase Catalysis

**DOI:** 10.1371/journal.pone.0163115

**Published:** 2016-09-19

**Authors:** Hasan Tükenmez, Helge Magnus Magnussen, Michael Kovermann, Anders Byström, Magnus Wolf-Watz

**Affiliations:** 1 Department of Molecular Biology, Umeå University, SE-901 87, Umeå, Sweden; 2 Department of Chemistry, Umeå University, SE-901 87, Umeå, Sweden; HUNGARY

## Abstract

Enzymes have evolved with highly specific values of their catalytic parameters *k*_cat_ and *K*_M_. This poses fundamental biological questions about the selection pressures responsible for evolutionary tuning of these parameters. Here we are address these questions for the enzyme adenylate kinase (Adk) in eukaryotic yeast cells. A plasmid shuffling system was developed to allow quantification of relative fitness (calculated from growth rates) of yeast in response to perturbations of Adk activity introduced through mutations. Biophysical characterization verified that all variants studied were properly folded and that the mutations did not cause any substantial differences to thermal stability. We found that cytosolic Adk is essential for yeast viability in our strain background and that viability could not be restored with a catalytically dead, although properly folded Adk variant. There exist a massive overcapacity of Adk catalytic activity and only 12% of the wild type *k*_cat_ is required for optimal growth at the stress condition 20°C. In summary, the approach developed here has provided new insights into the evolutionary tuning of *k*_cat_ for Adk in a eukaryotic organism. The developed methodology may also become useful for uncovering new aspects of active site dynamics and also in enzyme design since a large library of enzyme variants can be screened rapidly by identifying viable colonies.

## Introduction

Enzymes are remarkable bio-catalysts that can tremendously increase rates of otherwise slow cellular chemical reactions, thereby making them significantly faster than global processes, such as cell division, in living organisms Thus, for example, the estimated rate enhancement of the isomerization of (R)-mandelate to (S)-mandelate by mandelate racemase is 1.7x10^15^-fold [1]. Traditionally, catalytic parameters (*k*_cat_ and *K*_M_) are obtained from data gathered from *in vitro* experiments, such as spectroscopic observations of substrate depletion and accumulation of product molecules [2]. During the last decade NMR spectroscopy has added significant insights regarding the importance of dynamics (the time-dependent displacement of atomic coordinates) for enzymatic reaction cycles [3–8]. Several techniques, including NMR [9–11] and fluorescence microscopy [12], have also provided significant advances in analyses of proteins in their native environments inside living cells. This has enabled exploration of fundamental issues regarding, for instance, mechanisms allowing maintenance of enzymes’ functionality in the highly complex internal environments of living cells, where numerous variables could potentially affect their activities, such as macromolecular crowding, weak transient interactions and associated effects on translational diffusion [13].

However, the approaches mentioned above cannot address fundamental biological questions regarding the selection pressures responsible for evolutionary tuning of enzymes’ catalytic parameters, *k*_cat_ and *K*_M_. To address these questions we have developed an approach where we examine changes in relative fitness (obtained from growth rate constants) of yeast (*Saccharomyces cerevisiae*) cells expressing *Escherichia coli* adenylate kinase (Adk_eco_) variants with precise perturbations of the enzyme’s *k*_cat_ and *K*_M_ values for ATP turn-over ($K_{M}^{ATP}$). The approach is conceptually related to a previous study where yeast cell growth rates were analyzed in the context of ubiquitin stability [14]. It has been shown in a prokaryotic organism (*E*. *coli*) that there exist a large catalytic overcapacity of β–galactosidase activity [15] such that the relative fitness under limiting lactose concentrations is only affected when the catalytic activity of β–galactosidase is significantly impaired. In the present study we investigate the relative fitness of an eukaryotic organism in response to variations of the catalytic activity ofthe essential enzyme adenylate kinase (Adk).

**Fig 1:**
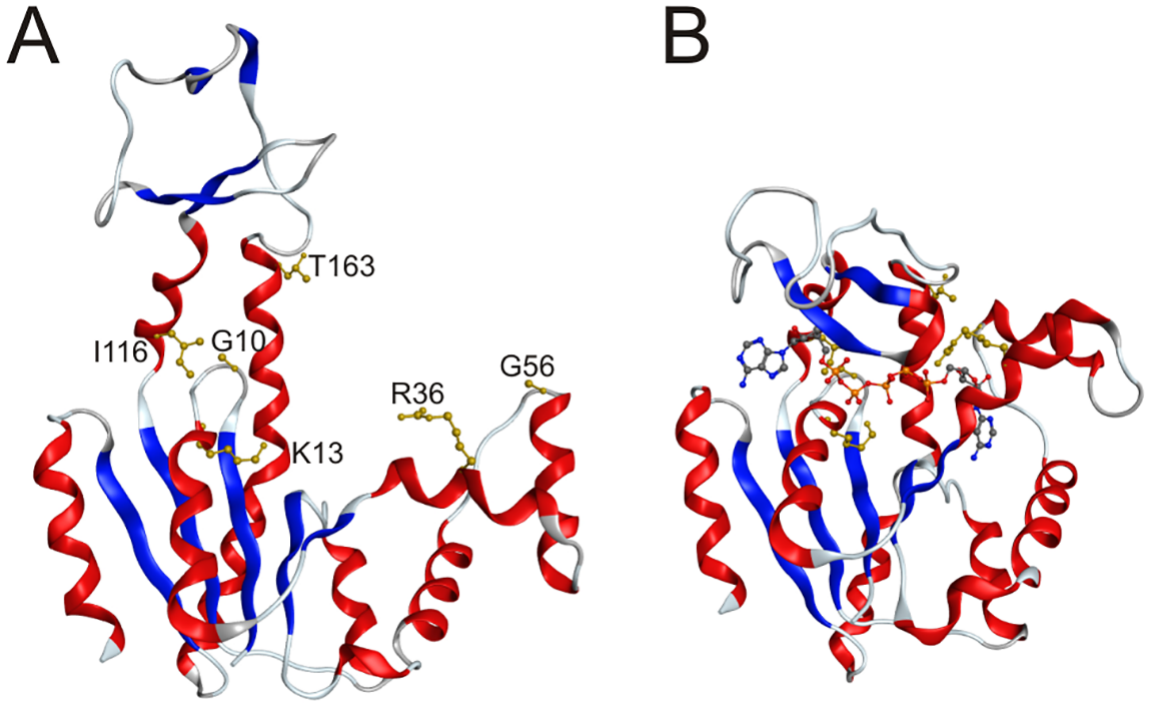
Structural dynamics in Adk_eco_ and positions of mutations. (**A**) Structure of the open and substrate free conformation of Adk_eco_ [16] (4AKE.pdb). Positions mutated in this study are indicated with ball and stick representations and colored in gold. (**B**) Crystallographic structure of Adk_eco_ in the closed and active conformation [17] (1AKE.pdb). The inhibitor Ap5A [29] is displayed with a ball and stick representation.

**Fig 2:**
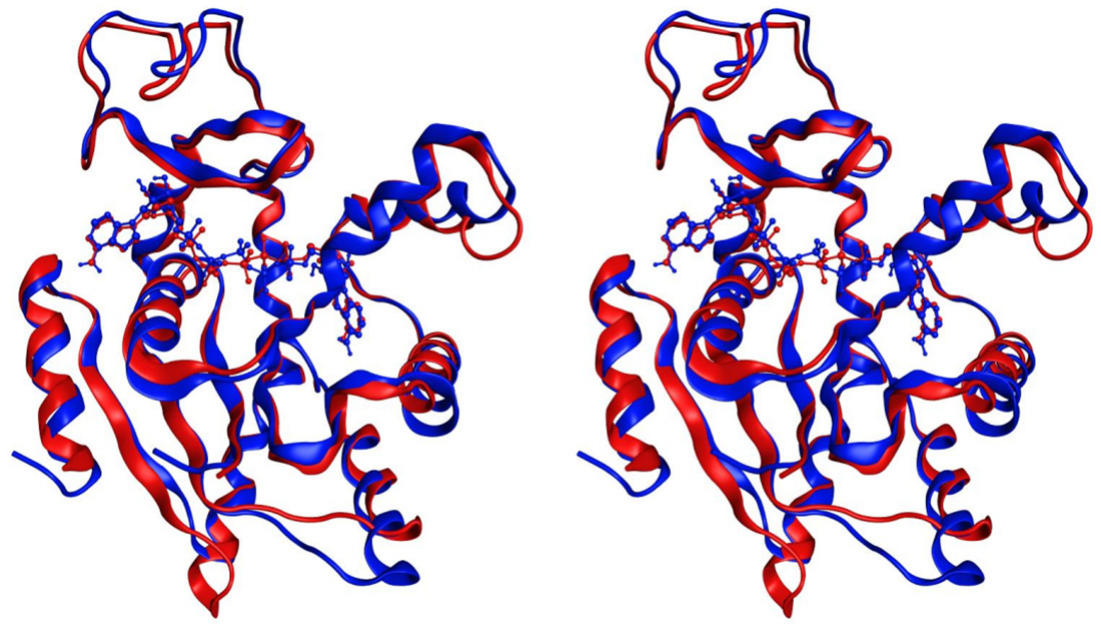
Structures of yeast and *E*. *coli* adenylate kinase in closed and active states. The stereo-view was made by superposition of C^α^ atoms of Adk1_yeast_ [26] (2AKY) and Adk_eco_ [17] (1AKE.pdb). The inhibitor Ap5A [29] is displayed with a ball and stick representation. Adk1_yeast_ and the corresponding Ap5A molecule is colored blue while Adk_eco_ with its corresponding Ap5A molecule is colored red.

Adk catalyzes the reversible and magnesium-dependent interconversion of ATP and AMP to two ADP molecules ($ATP+AMP_{\underset{k_{r}}{\leftarrow }}^{\overset{k_{f}}{\to }}2ADP$) and is required for maintenance of the cellular energy balance. The structural basis for Adk_eco_ has been extensively explored and there exist structures of substrate-free open [16] (Fig 1A) and also inhibitor bound closed structures [17] (Fig 1B). Likewise, the role of dynamics for the catalytic function of Adk_eco_ has been studied extensively. It has been shown with NMR [18, 19] and single molecule FRET experiments [20] [18] that the substrate-free enzyme transiently samples a “bound-like” structural state. Adk_eco_ is rate limited by substrate release and the microscopic explanation to this property is slow opening of the substrate binding domains in presence of bound substrate [7, 21]. Yeast was chosen as the eukaryotic model organism since robust tools are available for analyzing genes encoding mutated proteins [22]. Yeast cytosolic adenylate kinase (Adk1_yeast_) was selected as a target enzyme for the following reasons: absence of the enzyme is detrimental for yeast growth [23–25], crystallographic structures of the enzymes in yeast [26] and *E*. *coli* [17] have been determined (Fig 2), abundant information regarding catalytic properties of variants of the *E*. *coli* homologue (Adk_eco_) is available [19, 27], and there is substantial (47%) sequence identity between Adk_eco_ and Adk1_yeast_ [28], which is reflected in very similar three dimensional structures, with a root mean square deviation of 1.3 Å computed over C^α^ atoms (Fig 2). On basis of the above mentioned features of both yeast as a model organism and Adk as a model enzyme, we developed a yeast cell based approach to address the evolutionary constraints of Adk catalytic parameters in context of fitness of an eukaryotic organism.

## Experimental Procedures

### Strains, media and genetic procedures

The sources and genotypes of yeast and bacteria strains used in this study are listed in S1 Table. The yeast transformations [30], media and genetic procedures applied have been previously described [31]. The heterozygous strain UMY3969 (*ADK1/adk1*::*kanMX*) was generated from the diploid strain UMY3387 (*ADK1/ADK1*) by exchanging one of the *ADK1* open reading frames with the KanMX cassette. Strain UMY3969 was allowed to sporulate and tetrad analysis showed that *adk1Δ* strains were inviable. Diploid strain UMY3969 (*ADK1/adk1*::*kanMX*) was transformed with pRS316-*ADK1* followed by sporulation to obtain haploid strain UMY3974 (*adk1*::*kanMX* + pRS316-*ADK1*).

### Plasmid constructions

A SacI-BamHI fragment containing a wild type yeast *ADK1* open reading frame together with 600 bp upstream and 555 bp downstream regions was cloned into corresponding sites of either a *LEU2*-based low-copy number vector (pRS315) or a *URA3*-based low-copy number vector (pRS316), generating pRS315-*ADK1* and pRS316-*ADK1*. To clone the *E*. *coli adk* gene under control of the yeast *ADK1* promoter, we first PCR-amplified the 600 bp upstream region (as a SacI-XbaI fragment) and the 555 bp downstream region (as an XbaI-BamHI fragment) and cloned them together into the SacI and BamHI sites of the pRS315 vector, generating pRS315-Up-XbaI-Down. In this construct, the yeast *ADK1* open reading frame is exchanged with an XbaI restriction site. To obtain pRS315-*adk*_*eco*_, a DNA fragment encoding the wild-type *E*. *coli adk* open reading frame was PCR-amplified and exchanged with the XbaI site in pRS315-Up-XbaI-Down using the infusion cloning procedure (Clontech). To obtain *k*_cat_ mutant versions of the *E*. *coli adk* gene, pRS315-*adk*_*eco*_ was used as a template and mutations were introduced by PCR oligonucleotide-directed mutagenesis.

### Plasmid shuffling system

Strain UMY3974 (*MATa leu2-3*, *112 trp1-1 can1-100 ura3-1 ade2-1 his3-11*, *15 SSD1v-1 adk1*::*kanMX + pRS316-ADK1*) was transformed with pRS315 containing *E*. *coli adk* variants encoding Adk_eco_ with a range of *k*_cat_ values (Table 1). Transformants were selected on synthetic complete medium lacking uracil and leucine (SC-Ura-Leu) at 30°C. Three individual colonies from each transformation were incubated overnight in SC-Leu medium at 30°C. Serial dilutions of the saturated overnight cultures were plated on SC-Leu and SC-Leu+5-FOA (1 mg/ml) medium then incubated at 30°C. All mutants surviving the plasmid shuffling were streaked on SC-Ura and SC-Leu+5-FOA plates to confirm absence of the *URA3*-based pRS316-*ADK1* vector. Plasmids from these surviving mutants were isolated and sequenced to confirm the mutations.

**Table 1:** Nomenclature, catalytic parameters and melting temperatures of Adk variants.

Adk variant ^a^	substitution	*k*_cat_ (s^-1^)	Relative *k*_cat_	$K_{M}^{ATP}$ (μM)	*k*_cat_/$K_{M}^{ATP}$ (μM^-1^ s^-1^)	*T*_M_(°C)
Adk1_yeast_	-	520 ± 32	1.70	71 ± 18	7.29 ± 0.47	50.9±0.2
${\text{Adk}}_{\text{eco}}^{\text{1.00}}$	-	305 ± 12	1.00	72 ± 8	4.26 ± 0.17	55.4±0.2
${\text{Adk}}_{\text{eco}}^{\text{0.47}}$	T163C	143 ± 3	0.47	37 ± 2	3.90 ± 0.07	53.2±0.2
${\text{Adk}}_{\text{eco}}^{\text{0.20}}$	G10V	60 ± 3	0.20	411 ± 61	0.14 ± 0.01	49.3±0.2
${\text{Adk}}_{\text{eco}}^{\text{0.12}}$	G56C	36 ± 0.5	0.12	34± 2	1.04 ± 0.01	49.5±0.2
${\text{Adk}}_{\text{eco}}^{\text{0.06}}$	R36A	19 ± 0.2	0.06	193± 7	0.10 ± 0.00	53.4±0.2
${\text{Adk}}_{\text{eco}}^{\text{0.04}}$	I116G	11 ± 5 ^b^	0.04	27.0 ± 5 ^b^	0.41 ± 0.19 ^b^	53.6±0.2
${\text{Adk}}_{\text{eco}}^{\text{0.007}}$	R36S + 11aa ^c^	2.0 ± 0.1	0.007	127± 30	0.02 ± 0.00	49.6±0.2
${\text{Adk}}_{\text{eco}}^{\text{0.0002}}$	K13Q	0.06 ^d^	0.0002	1400.0 ^d^	0.00 ^d^	54.4±0.2

^a)^ *E*. *coli* Adk variants are shown as “${\text{Adk}}_{\text{eco}}^{\text{x}}$”, where “x” indicates *k*_*cat*_ values relative to the *k*_*cat*_ value of wild-type *E*. *coli* ${\text{Adk}}_{\text{eco}}^{\text{1.00}}$.

^b)^ Data from Olsson *et al*.

^c)^ The insertion of 11 amino acids corresponds to the sequence “STGDMLSAAVK” inserted between residues Lys40 and Ser41.

^d)^ Data from Reinstein *et al 1990*.

The errors reported for the catalytic parameters *k*_cat_, $K_{M}^{ATP}$ and *k*_cat_/$K_{M}^{ATP}$ are corresponding to standard deviations from three technical replicates. The errors for the *T*_m_ values are based on a conservative estimate of the experimental uncertainty from multiple evaluations of the *T*_m_ of $Adk_{eco}^{1.00}$.

### Protein extraction and western blotting

Cells were cultivated logarithmically at 20°C until their optical density reached 0.5 at 600 nm and 5 OD-units of cells were harvested using a previously described TCA protein extraction procedure [32]. Proteins were separated by 12% SDS-PAGE and transferred to nitrocellulose membranes. To detect Adk_eco_ proteins, a rabbit polyclonal anti-Adk_eco_ (Karuso, 14EF 34) antibody was used (Agrisera, Sweden). Actin was detected using a mouse anti-Act1 antibody (Thermo Scientific). Protein levels were quantified using ImageJ software. Act1 protein levels were used as loading controls and protein levels of the Adk_eco_ variants were normalized according to wild-type ${\text{Adk}}_{\text{eco}}^{\text{1.00}}$.

### Protein expression and purification

Adk variants were produced, ^15^N-enriched and purified as previously described [7]. ${\text{Adk}}_{\text{eco}}^{\text{0.007}}$ and ${\text{Adk}}_{\text{eco}}^{\text{0.0002}}$ did not bind to Blue Sepharose so the flow-through was loaded on a Q-Sepharose column and these variants were eluted as previously described [27].

### Coupled ATPase assay

Adk activity was quantified at 20°C in the direction of ADP formation with a coupled ATPase assay as outlined previously [33]. The assay couples ADP production to oxidation of NADH through the activity of pyruvate kinase and lactate dehydrogenase. Pyruvate kinase catalyzes the conversion of ADP and phosphoenolpyruvate to pyruvate and ATP. Lactate dehydrogenase in turn catalyzes the conversion of pyruvate and NADH to lactate and NAD+. The assay was performed in a buffer consisting of 80 mM KCl, 2mM MgCl_2_ and 100 mM Tris at pH 7.5. The constituents used for the coupled reactions were phosphoenolpyruvate present at 0.4 mM and NADH present at 0.2 mM. The AMP concentration was held constant at 300 μM which is well above the $K_{M}^{AMP}$ value of Adk_eco_ that previously was found to be 26 μM [27]. 1.1–43 nM of Adk variants were used in the reactions. The consumption of NADH was quantified by following the change in absorbance at 340 nm and by using an extinction coefficient of 6220 M^-1^ cm^-1^. The corresponding time-dependent ATP consumption (*V* in S1 Fig) is related to the half the change in NADH concentration since two ADP molecules is produced for each ATP molecule consumed. Care was taken to verify that sufficient amounts of pyruvate kinase and lactate dehydrogenase were present such that the NADH oxidation was limited by Adk catalysis. Reagents were purchased from Sigma-Aldrich. Catalytic parameters, *k*_cat_, and $K_{M}^{ATP}$, were obtained through fits of initial velocities (*V*) in response to variation of the ATP concentration ([S] in Eq 1) to the Michaelis-Menten equation:

$$V=\frac{V_{max}\left[\text{S}\right]}{K_{M}+\left[\text{S}\right]}$$ (1)

Since AMP is held at a constant concentration the reported $K_{M}^{ATP}$ values should be treated as apparent *K*_M_ values. The catalytic parameters of the variants I116G and K13Q were taken from the previous studies [34] and [35], respectively.

### NMR spectroscopy

NMR spectra were acquired on a Bruker 850 MHz Avance III HD equipped with a 5 mm TCI cryoprobe (Bruker Biospin) or a Bruker 500 MHz Avance III equipped with a 5 mm TBI probe. The samples contained 100–400 μM ^15^N-labeled Adk in a buffer consisting of 10% (v/v) ^2^H_2_O, 50 mM NaCl and 30 mM MOPS at pH 6.0.

### Circular dichroism

Far ultraviolet circular dichroism (CD) experiments were performed on a Jasco J-810 spectropolarimeter. Thermal unfolding was followed by monitoring the CD signal at 220 nm in a 1 mm cuvette with a scan rate of one degree min^-1^. Protein concentrations in the CD experiments were 15 μM in a buffer consisting of 10 mM sodium phosphate and 50 mM NaCl at pH 7.0. Melting temperatures (*T*_M_) were quantified with non-linear fits (Microcal Origin) of CD-data to a two-state transition [36].

## Results and Discussion

### Design of Adk variants

**Fig 3:**
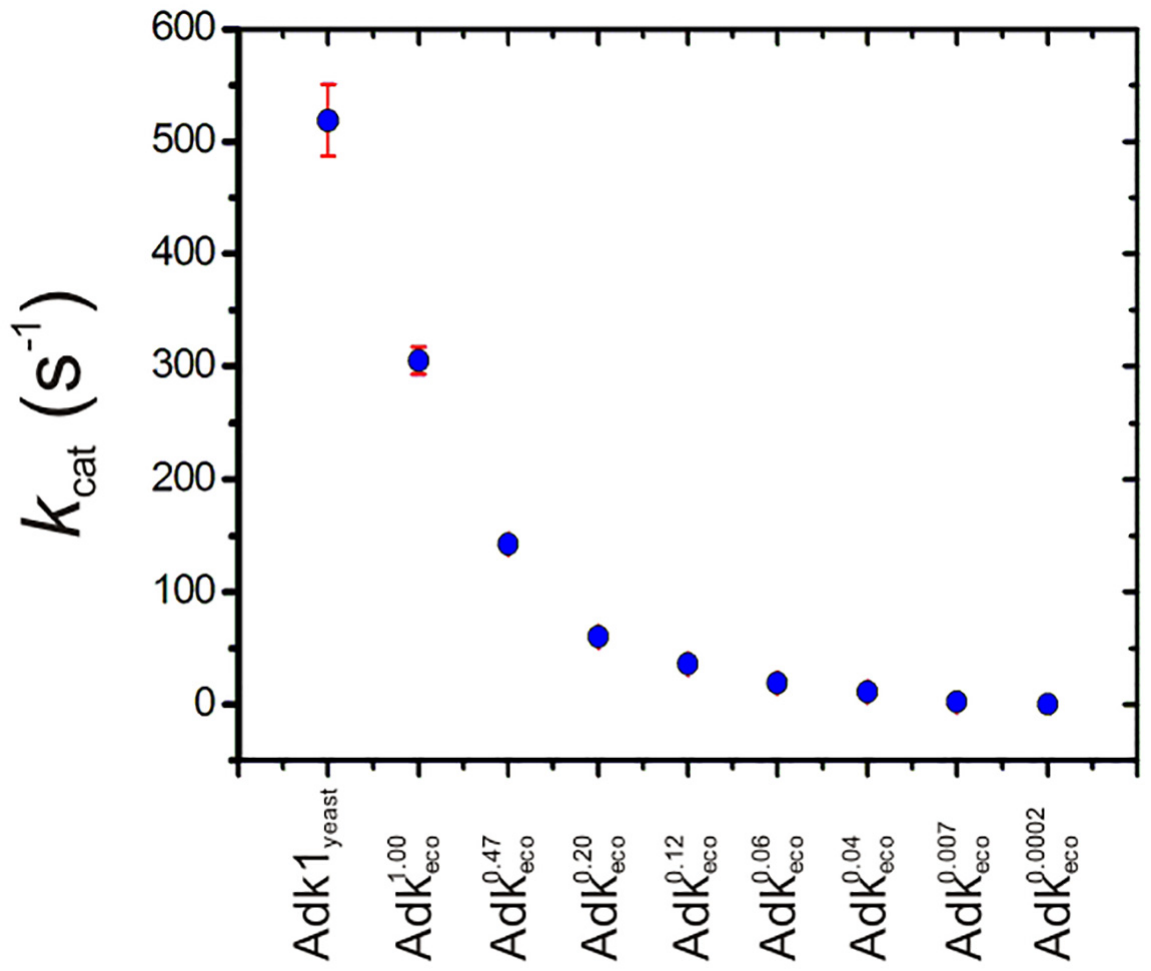
Catalytic activity of Adk variants at 20°C. Adk variants analyzed were designed to have a broad coverage of *k*_cat_ which is illustrated by a display of *k*_cat_ from Table 1 vs the corresponding Adk variant.

To enable a test of yeast growth rates in response to variation in the *k*_cat_ of Adk we designed a set of mutations that has a logarithmic coverage of this crucial catalytic parameter (Fig 3). Activity was determined at 20°C and since the only variant that displayed reduced yeast growth rates at 30°C (see below) was the essentially catalytically dead Adk_eco_ variant K13Q, 20°C is an appropriate temperature for activity measurements. First we determined *k*_cat_ and $K_{M}^{ATP}$ of yeast Adk1 and compared these values to published values of Adk_eco_ parameters. It was found that the $K_{M}^{ATP}$ values are very similar for the two enzymes (Table 1 and S1 Fig) but that there is a sizable difference in the *k*_cat_ values that are of 305±12 s^-1^ for Adk_eco_ and 520±32 s^-1^ for Adk1_yeast_. Thus replacement of Adk1 for Adk_eco_ in the experimental approach described below will in fact represent a data point where *k*_cat_ is 59% compared to the yeast wild-type. The remaining variation in *k*_cat_ relative to yeast Adk1 was accomplished through point mutations and in one case an insertion of 11 amino acids into Adk_eco_. For clarity, the variants of Adk_eco_ are denoted ${\text{Adk}}_{\text{eco}}^{\text{x}}$, where “x” indicates the *k*_cat_ value relative to that of wild-type *E*. *coli* Adk_eco_ (hereafter denoted ${\text{Adk}}_{\text{eco}}^{\text{1.00}}$). For instance Adk_eco_, with a T163C amino acid substitution (for which the *k*_cat_ is 47% of the ${\text{Adk}}_{\text{eco}}^{\text{1.00}}$ value) is denoted ${\text{Adk}}_{\text{eco}}^{\text{0.47}}$. The identity of the mutations and the *k*_cat_ values associated with the Adk_eco_ variants used in this study are summarized in Table 1. Displays of the kinetic traces for the unique variants analyzed in this study are displayed in S1 Fig.

### Structure and stability of Adk variants

**Fig 4:**
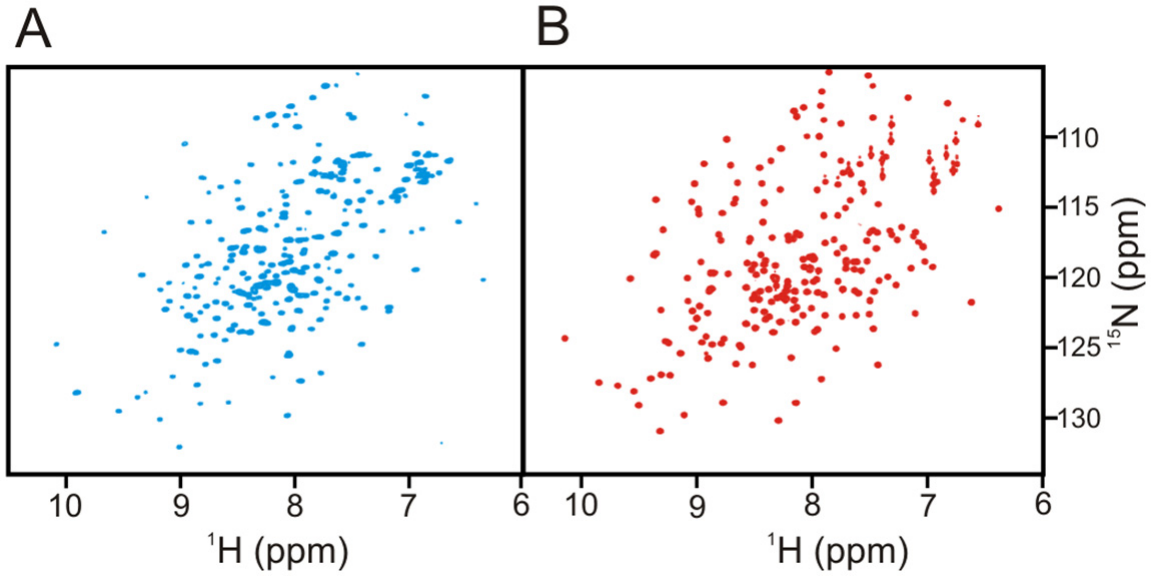
NMR spectra of yeast Adk1 and *E*. *coli* Adk. **(A)** ^1^H-^15^N HSQC spectrum of Adk1_yeast_ **(B)** ^1^H-^15^N HSQC spectrum of Adk_eco_. The spectra were acquired at 20°C and show that both enzymes are properly folded at the conditions used in this study.

**Fig 5:**
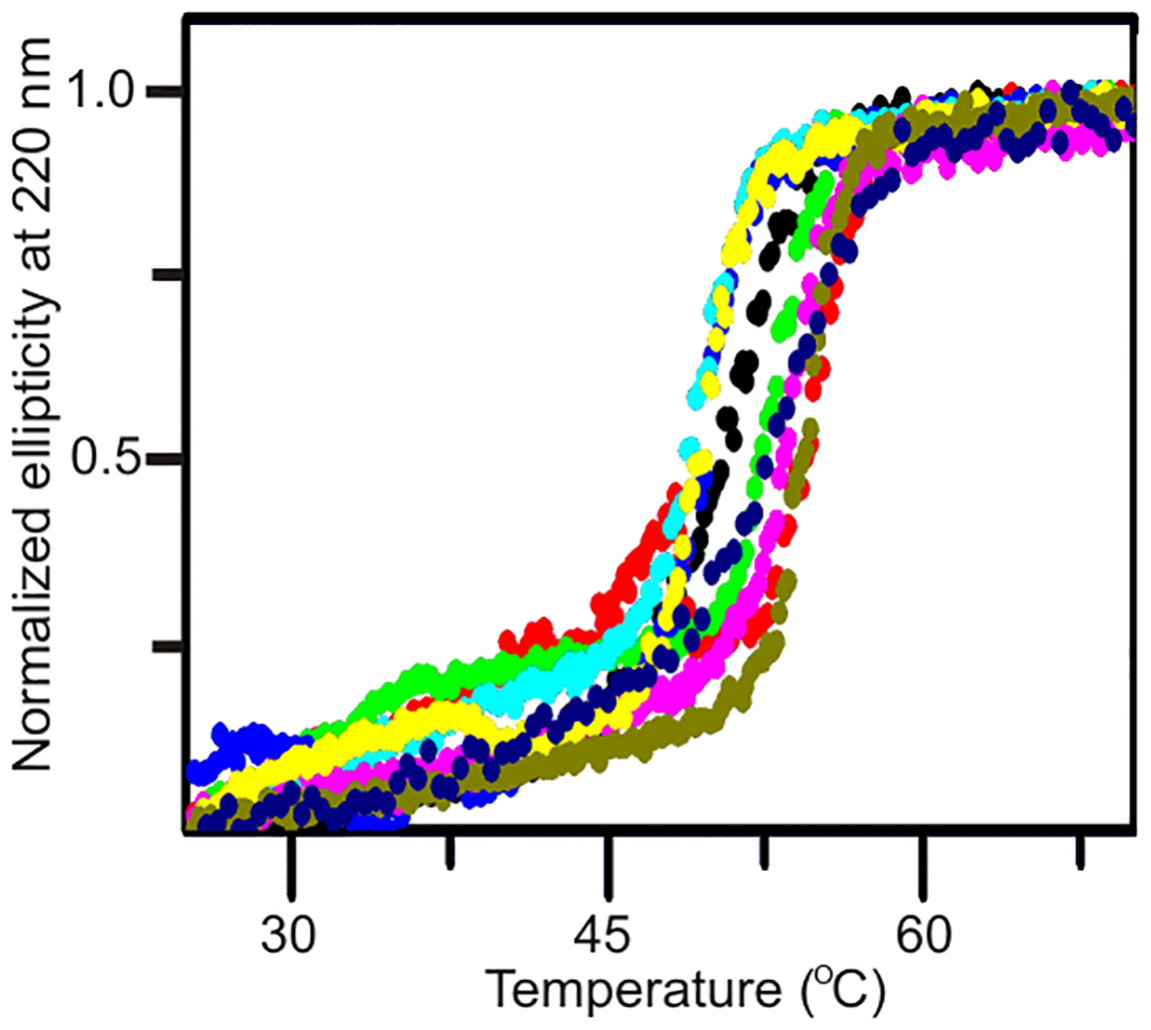
Thermal stability of Adk variants. The thermal stability of Adk1_yeast_ (black), ${\text{Adk}}_{\text{eco}}^{\text{1.00}}$ (red), ${\text{Adk}}_{\text{eco}}^{\text{0.47}}$ (green), ${\text{Adk}}_{\text{eco}}^{\text{0.20}}$ (blue), ${\text{Adk}}_{\text{eco}}^{\text{0.12}}$ (turquoise), ${\text{Adk}}_{\text{eco}}^{\text{0.06}}$ (pink), ${\text{Adk}}_{\text{eco}}^{\text{0.04}}$ (purple), ${\text{Adk}}_{\text{eco}}^{\text{0.007}}$ (yellow) and ${\text{Adk}}_{\text{eco}}^{\text{0.0002}}$ (olive) was quantified by observing normalized circular dichroism signals at a wavelength of 220 nm as a function of temperature. The data are displayed assuming a two-state unfolding model. Associated melting temperatures (*T*_M_) are displayed in Table 1.

To interpret the results purely in terms of effects of *k*_cat_ and $K_{M}^{ATP}$ perturbations it was important to confirm that the tested mutations did not cause potentially confounding effects on the structural integrity and stability of the variants. Therefore, we investigated the structural integrity of all Adk variants used in this study with two-dimensional heteronuclear high-resolution NMR spectroscopy. All variants display well-dispersed ^1^H-^15^N HSQC spectra as shown for Adk_eco_ and Adk1_yeast_ in Fig 4, showing that the mutations (S2 & S3 Figs) do not cause any global structural perturbations to the enzymes and that the variants only have local structural differences related to the individual amino acid replacements (and in one case insertion of 11 amino acids). From a stability perspective it was essential for the melting temperatures (*T*_M_) to be well above the cultivation temperatures to be used in the yeast growth experiment (20°C– 30°C) to ensure that the proteins were properly folded under the experimental conditions. Circular dichroism spectroscopy demonstrated that the *T*_M_ of all variants are well above the temperature interval used in the growth experiments (Fig 5). Thus, all variants fulfilled the structural integrity and thermal stability criteria, and the tested mutational perturbations can be considered as clean variations of *k*_cat_ and $K_{M}^{ATP}$ that do not affect the global structure and stability of the enzyme.

### Analysis of yeast fitness

**Fig 6:**
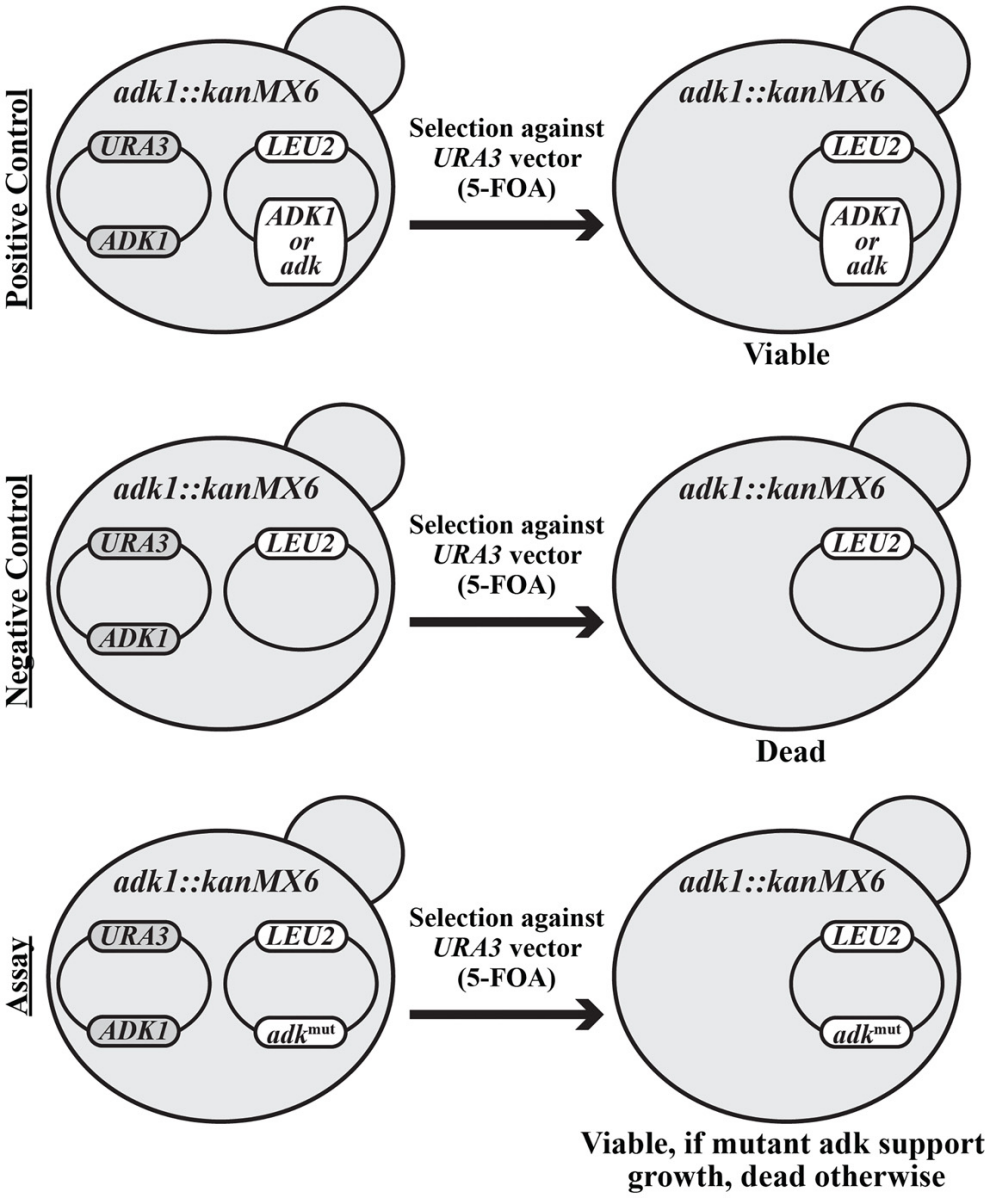
Yeast plasmid shuffling assay system. The yeast *ADK1* open reading frame was exchanged with the KanMX cassette. Viability of the resulting strain depends on the presence of a wild-type yeast *ADK1* gene in a low-copy number *URA3*-based vector, pRS316. A second low-copy number *LEU2*-based vector was used to introduce different alleles of the *E*. *coli adk* gene into this strain. Thus, a strain harboring both the *URA3* plasmid (wild-type yeast *ADK1* gene) and the *LEU2* plasmid (mutated *E*. *coli adk* gene) can be obtained. If such a strain is plated on medium containing 5-FOA, the *URA3* vector will be counter-selected as the *URA3* gene product converts 5-FOA to a toxic compound [22]. Thus, this plasmid shuffling procedure can reveal the phenotype conferred by a mutated *E*. *coli adk* gene located in the *LEU2* plasmid.

**Fig 7:**
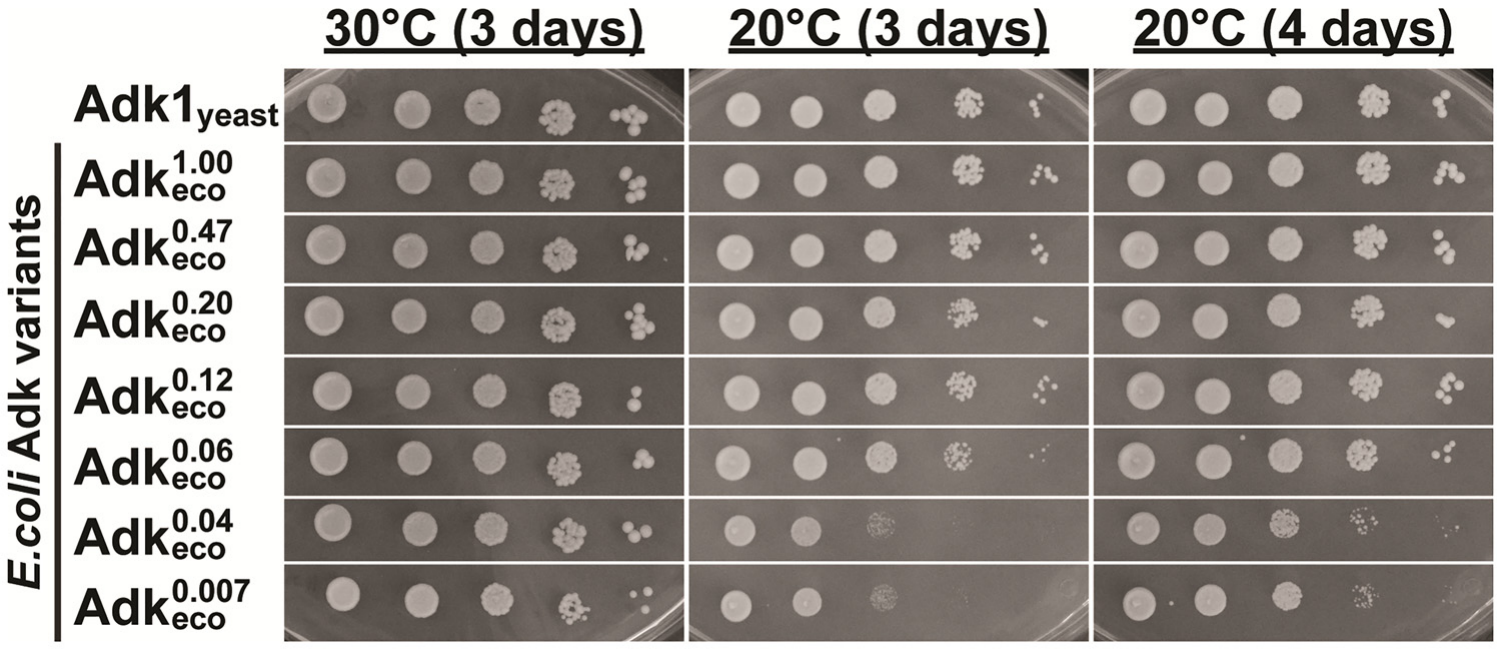
Serial dilution growth assays at 30°C and 20°C. Yeast *adk1Δ* cells expressing Adk1_yeast_ or indicated variants of Adk_eco_ proteins were serially diluted, spotted on SC-Leu plates, and incubated at 30°C and 20°C for 3–4 days.

Here we have developed a yeast cell based approach to address the relevance of the catalytic parameters *k*_cat_ and $K_{M}^{ATP}$ of adenylate kinase (Adk) for the fitness of yeast expressing Adk variants. Depending on the strain background, *adk1Δ* yeast strains are either very sick or inviable [23–25]. In the present study we first showed that in our yeast strain background a knock-out of the *ADK1* gene is lethal and can be rescued by a plasmid-borne wild-type yeast *ADK1* (see strains, media and genetic procedures in experimental procedures). To study the cell growth responses to perturbations of adenylate kinase *k*_cat_ and $K_{M}^{ATP}$ parameters in this system, we expressed specifically mutated variants of Adk_eco_. The rationale for using Adk_eco_ rather than Adk1_yeast_ for this purpose was that more information is available about Adk_eco_ variants and their catalytic properties. Thus, genes encoding Adk_eco_ variants with perturbations in both *k*_cat_ and $K_{M}^{ATP}$ were introduced to yeast with a plasmid shuffling system [22] illustrated and explained in Fig 6. According to a coupled ATPase assay, there were no significant differences in $K_{M}^{ATP}$ values between wild-type Adk_eco_ and Adk1_yeast_ at 20°C, but the *k*_cat_ of Adk_eco_ (305±12 s^-1^) is 59% relative to that of Adk1_yeast_ (520±32 s^-1^) (Table 1). Despite the differences in catalytic turn-over, the growth rates of *adk1Δ* yeast cells supplemented with wild-type yeast *ADK1* or *E*. *coli adk* genes were identical, demonstrating that the *E*. *coli adk* gene could functionally exchange the yeast *ADK1* gene (Table 2). Initially, we compared the growth properties of yeast cells expressing Adk variants in a serial dilution assay, and detected no growth defects in cells expressing the Adk_eco_ variants at 30°C, the optimal growth temperature for yeast (Fig 7). Only the K13Q mutant (${\text{Adk}}_{\text{eco}}^{\text{0.0002}}$) with a *k*_cat_ of 0.06 s^-1^ at 20°C (basically catalytically dead variant) failed to rescue the knockout. Next, we tested effects of the perturbations in response to stress by decreasing the growth temperature to 20°C. At 20°C growth of cells expressing ${\text{Adk}}_{\text{eco}}^{\text{0.06}}$ and ${\text{Adk}}_{\text{eco}}^{\text{0.007}-\text{0.04}}$ were slightly and strongly impaired, respectively (Fig 7). The *k*_cat_ values of these mutants are 19, 11 and 2 s^-1^, respectively. The variant with lowest activity and with a growth that cannot be distinguished to that of yeast expressing Adk1_yeast_ is ${\text{Adk}}_{\text{eco}}^{\text{0.12}}$ that has a *k*_cat_ of 36 s^-1^. Apparently, the stress imposed by the 10°C reduction of growth temperature results in a significant increase in the *k*_cat_ value required for optimal growth. It should be noted that the *k*_cat_ values were measured at 20°C and that some of the effect observed may be due to differences in the *k*_cat_ values at 20 and 30°C. The temperature dependency of *k*_cat_ of Adk_eco_ has been quantified previously [21] and there exist a 2 fold difference in activity between 20 and 30°C. Assuming that the temperature dependency is similar for the mutant forms of Adk_eco_ studied here it is likely that at least, a part of the overcapacity of adenylate kinase is required for adaptation of yeast to stress conditions. On the basis of the comparison of growth at 20°C and 30°C, subsequent liquid culture experiments were performed at 20°C.

**Table 2:** Expression levels, growth rate constants, relative fitness and apparent *k*_cat_ values of Adk_eco_ variants at 20°C.

Adk variant	Protein levels ^a^	Growth rate constants (h^-1^)	Relative Fitness ^b^	${\text{k}}_{\text{cat}}^{\text{app}}$ (s^-1^) ^c^
Adk1_yeast_	n.d. ^d^	0.30 ± 0.01	1.0 ±0.05	n.d. ^e^
${\text{Adk}}_{\text{eco}}^{\text{1.00}}$	1.00 ± 0.00	0.30 ± 0.01	1.0 ±0.05	305 ± 12
${\text{Adk}}_{\text{eco}}^{\text{0.47}}$	1.04 ± 0.10	0.30 ± 0.01	1.0 ±0.05	149 ± 15
${\text{Adk}}_{\text{eco}}^{\text{0.20}}$	1.93 ± 0.19	0.30 ± 0.02	1.0 ±0.07	116 ± 13
${\text{Adk}}_{\text{eco}}^{\text{0.12}}$	1.92 ± 0.19	0.29 ± 0.01	0.97 ±0.05	69± 6.9
${\text{Adk}}_{\text{eco}}^{\text{0.06}}$	2.36 ± 0.12	0.27 ± 0.02	0.90 ±0.07	45 ± 2.3
${\text{Adk}}_{\text{eco}}^{\text{0.04}}$	4.52 ± 0.08	0.20 ± 0.01	0.67±0.04	50 ± 23
${\text{Adk}}_{\text{eco}}^{\text{0.007}}$	4.60 ± 0.21	0.21 ± 0.01	0.7 ±0.04	9 ± 0.5
${\text{Adk}}_{\text{eco}}^{\text{0.0002}}$	n.d. ^e^	n.d. ^e^	n.d. ^e^	n.d. ^e^

^a^ Protein levels were determined by quantification of band intensities in western blots and normalized to wild-type *E*. *coli* ${\text{Adk}}_{\text{eco}}^{\text{1.00}}$.

^b^ Relative fitness is the ratio of growth rates of yeast cells harboring Adk_eco_ variants to cells harboring yeast Adk1.

^c^ Apparent *k*_cat_ represents the normalized *k*_cat_ values with the relative expression levels according to Eq 2.

^d^ Adk1_yeast_ cannot be detected by rabbit polyclonal anti-Adk_eco_ antibody.

^e^ Protein levels and cell growth rate constants cannot be determined as the *adk1Δ* strain expressing ${\text{Adk}}_{\text{eco}}^{\text{0.0002}}$ is not viable.

Error bars for protein levels and growth rate constants indicate standard deviations obtained from three independent biological replicates.

**Fig 8:**
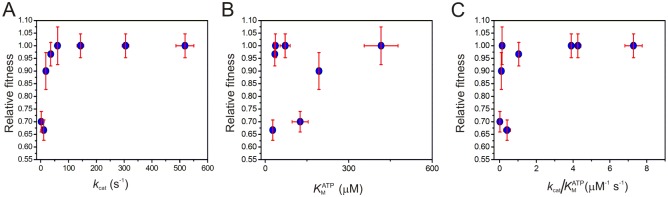
Dependencies of relative fitness on Adk catalytic parameters *k*_cat_, $K_{M}^{ATP}$ and *k*_cat_/$K_{M}^{ATP}$. **(A)** Relative fitness plotted versus *k*_cat_. **(B)** Relative fitness plotted versus the Michaelis constant (*K*_M_). **(C)** Relative fitness plotted versus the specificity constant (*k*_cat_/$K_{M}^{ATP}$). Cells were cultivated for growth rate measurements at 20°C. Catalytic parameters (*k*_cat_ and $K_{M}^{ATP}$) were obtained from a coupled ATPase assay [33]. Error bars for growth rate constants indicate standard deviations obtained from three independent biological replicates. Error bars for Adk catalytic parameters *k*_cat_ and $K_{M}^{ATP}$ indicate standard deviations of three technical replicates.

**Fig 9:**
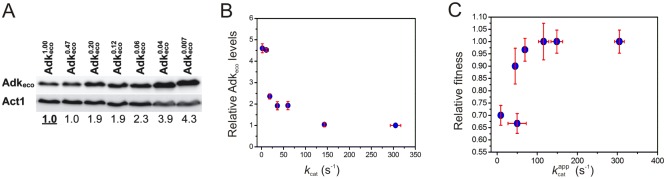
Effect of Adk_eco_ expression levels in yeast cells. **(A)** Western blot analysis of Adk_eco_ proteins expressed from the pRS315 vector in *adk1Δ* yeast cells and detected by a rabbit polyclonal anti-Adk_eco_ antibody. Endogenous Act1p was used as a loading control. Protein levels of the Adk_eco_ variants were normalized with respect to wild-type ${\text{Adk}}_{\text{eco}}^{\text{1.00}}$ protein levels (Table 2). **(B)** Increases in Adk_eco_ protein levels correlate with reductions in catalytic activity (*k*_cat_). **(C)** Relative fitness plotted versus $k_{\text{cat}}^{\text{app}}$ (*i*.*e k*_cat_ normalized with respect to Adk_eco_ protein levels according to Eq 2). Cells were cultivated at 20°C for western blots. Error bars for protein levels indicate standard deviations obtained from three independent biological replicates.

As indicated above, yeast cell growth is sensitive to the perturbations of the catalytic activity of adenylate kinase when the cells are subjected to stress by growth at the sub-optimal temperature 20°C. To quantify the influence of the quantified values of *k*_cat_, $K_{M}^{ATP}$ and the specificity constant *k*_cat_/$K_{M}^{ATP}$, the relative fitness of yeast cells expressing all tested Adk variants were quantified at 20°C in liquid cultures (Table 2). The relative fitness is defined as the ratio between the growth rate of yeast transformed with an Adk variant divided by the growth rate of yeast transformed with yeast Adk1 [15]. Relative fitness were then plotted against *k*_cat_, $K_{M}^{ATP}$ and *k*_cat_/$K_{M}^{ATP}$, to identify whether perturbation of *k*_cat_ or $K_{M}^{ATP}$ was responsible for the growth impairment observed in the serial dilution assay at 20°C. When the relative fitness was displayed against *k*_cat_ values (Fig 8A) the first conclusion was that the fitness was identical for cells expressing either yeast Adk1 or *E*. *coli* Adk. Since the *k*_cat_ value of the *E*. *coli* enzyme is 59% (Table 1) relative to the yeast enzyme it is immediately evident that there is substantial overcapacity in the Adk catalytic power in yeast cells. Clear impairment of the relative fitness is only observed when the *k*_cat_ value is below 60 s^-1^ and further reduction in *k*_cat_ results in successive slower growth rates. Overall the shape of the plot resembles a saturation curve and the relative fitness of yeast expressing Adk_eco_ variants displaying as little as 7% catalytic activity compared to wild-type yeast Adk1 (12% of wild type ${\text{Adk}}_{\text{eco}}^{\text{1.00}}$) were indistinguishable from those expressing Adk1_yeast_. In contrast, there was no obvious correlation between the variants’ relative fitness and $K_{M}^{ATP}$ values when $K_{M}^{ATP}$ is varied in the interval 34–411 μM (Fig 8B). Not surprisingly a display of relative fitness against *k*_cat_/$K_{M}^{ATP}$ ratios showed a functional dependency that is similar to that observed when *k*_cat_ was analyzed (Fig 8C). Thus, the lack of correlation for $K_{M}^{ATP}$ is superseded by the correlation to *k*_cat_ when *k*_cat_/$K_{M}^{ATP}$ is displayed. Taken together, it is apparent that the relative fitness in the experiment is predominantly dependent on the *k*_cat_ values of the Adk variants encoded by the introduced plasmids. An important aspect for the interpretation of the *in vivo* experiments are the expression levels of the various Adk_eco_ variants since a variation in these levels potentially can affect the conclusions drawn. Protein expression levels were quantified with western blot analysis using a polyclonal antibody raised against Adk_eco_ (Fig 9A). Increased Adk_eco_ protein levels were observed for variants where *k*_cat_ is below 60 s^-1^ (Fig 9B). Thus the expression levels were increased for variants that also displayed a growth rate impairment. Hence, the yeast cells seem to compensate the perturbation to the growth rate by an upregulation of the Adk_eco_ expression levels. These increases in expression levels were presumably due to either *k*_*cat*_-dependent upregulation of protein expression or selection of cells with a higher copy number of plasmids harboring the *E*. *coli adk* mutant gene. The differences in expression levels do however not change the main observation that cells do have optimal growth rates even with severe reduction of *k*_*cat*_. This inference is illustrated with a display of relative fitness against apparent *k*_cat_ values ($k_{\text{cat}}^{\text{app}}$) (Fig 9C), where $k_{\text{cat}}^{\text{app}}$ corresponds to *k*_cat_ normalized with the relative expression levels (Table 2) according to Eq 2.

$$k_{\text{cat}}^{\text{app}}\;=\;k_{\text{cat}}\;*\;[\text{relative}\;\text{expression}\;\text{levels}]$$ (2)

## Conclusions

Here we report an approach that enables *in vivo* analysis of effects of perturbations of adenylate kinase catalytic parameters in yeast cells. In the experiments growth rates of cells were quantified in response to Adk_eco_ variants with mutations that “cleanly” perturb the targeted parameters (*k*_cat_ and $K_{M}^{ATP}$) without compromising the enzyme’s structural integrity or thermal stability. First we showed that cytosolic Adk is required for yeast viability in our strain background, and that a catalytically dead but properly folded variant (K13Q) could not restore viability. These results supports that the relative fitness of the yeast strains is depending on the level of Adk activity. The intracellular ATP concentrations in yeast cells are in the low millimolar range [37–39]. The Michaelis constant is related to the dissociation constant (*K*_d_) for a given substrate, and it has been shown that the *K*_d_ of ATP binding to Adk_eco_ is 50 μM [40] which is close to the value of $K_{M}^{ATP}$ determined here (~70 μM). With $K_{M}^{ATP}$ values used as a proxy for ATP binding affinity it is, in fact, expected that all Adk variants used in this study should be saturated with ATP inside yeast cells since the $K_{M}^{ATP}$ values ranging from 34 to 417 μM are below the expected cellular ATP levels. This inference was corroborated with the cell growth experiments since no correlation between relative fitness and $K_{M}^{ATP}$ values was observed (Fig 8B). Thus, from a standpoint of $K_{M}^{ATP}$, the Adk variants are all fully functional in the cellular milieu and the growth defects observed in the experiments can be attributed to variations in *k*_cat_ (determined at 20°C). It was found that yeast cell growth at the optimal temperature (30°C) was not affected by the mutations (except for ${\text{Adk}}_{\text{eco}}^{\text{0.0002}}$ with a *k*_cat_ of 0.06 s^-1^ that was unable to rescue the inviable phenotype), which is remarkable as the *k*_cat_ of one of the mutants is only 0.4% compared to wild-type Adk1_yeast_. In contrast, in response to external stress by growth at a sub-optimal temperature of 20°C the relative fitness of yeast was impaired when the *k*_cat_ value was less than 7% of the Adk1_yeast_ value. Taken together the data show that the *k*_cat_ value of Adk1_yeast_ is well above the threshold value required for cell growth under optimal and sub-optimal conditions and that there is substantial overcapacity in the catalytic turn over by Adk in yeast cells. A similar functional dependency of relative fitness with a massive catalytic overcapacity has been observed in *E*. *coli* for the enzyme β-galactosidase [15, 41]. Thus, both the present study in a eukaryotic organism and the cited β-galactosidase studies in a prokaryotic organism indicate that only a fraction of evolved enzymatic activity may be required for optimal cell growth under laboratory conditions. The data presented here show that, at least a part of the overcapacity in catalytic power is required for organisms to survive external stress conditions that may apply to organisms in their natural habitats. There exist other ways of inflicting stress conditions to yeast in laboratory growth experiments and examples thereof are; oxidative stress, and nutritional stress. For the conceptual discovery here temperature was chosen since it is a parameter that can be accurately controlled and no additional variables such as nutritional uptake or intracellular concentrations (of for instance hydrogen peroxide in oxidative stress experiments) needs to be considered. Additionally, the plasmid shuffling system developed here is a useful platform in order to promote novel discoveries in Adk enzymology. This can in principle be performed by searching for intragenic suppressor mutations that can revert/save an inviable phenotype dependant on mutation of key catalytic residues. Intragenic suppressor mutations may bypass the effect of the mutation leading to inviability and this bypass effect can generate novel information on, for instance, the plasticity of active sites. A second application to the method lies within enzyme design, here it is possible to make use of the fact that yeast can survive with a very low *k*_cat_ value at 30°C (Fig 7). One useful experiment that contains significant information on design would be to evolve adenylate kinase activity from an unrelated ATP binding enzyme. Random mutation of the gene encoding this scaffold enzyme followed by transformation into the yeast plasmid shuffling system would generate viable colonies only if adenylate kinase activity has evolved.

## Supporting Information

S1 FigEnzyme kinetics with ATP as variable substrate.The velocity of ADP production (*V* with unit M s^-1^) is scaled by the enzyme concentration (*V*/[Adk]) to obtain the displayed parameter of the y-axis with the unit s^-1^. The assays were performed with an AMP concentration held constant at 300 μM at 20°C. Error bars are obtained from the standard deviation resulting from three technical replicates. Displayed are the unique variants analyzed in this study and also ${\text{Adk}}_{\text{eco}}^{\text{1}.00}$ (**A**) Adk1_yeast_. (**B**) ${\text{Adk}}_{\text{eco}}^{\text{1.00}}$ (**C**) ${\text{Adk}}_{\text{eco}}^{\text{0.47}}.\;$(**D**)$\;{\text{Adk}}_{\text{eco}}^{\text{0.20}}$. (**E**) ${\text{Adk}}_{\text{eco}}^{\text{0.12}}$. (**F**)$\;{\text{Adk}}_{\text{eco}}^{\text{0.06}}$. (**G**)$\;{\text{Adk}}_{\text{eco}}^{\text{0.007}}$.(DOCX)

S2 Fig ^1^H-^15^N HSQC spectra of Adk_eco_ variants. (**A**) T163C. (**B**) G10V. (**C**) G56C. (**D**) R36A.
(DOCX)

S3 Fig ^1^H-^15^N HSQC spectra of Adk_eco_ variants. (**A**) I116G. (**B**) R36S+11a.a. (**C**) K13Q.
(DOCX)

S1 Table Yeast strains used in this study. (DOCX)
