# Supplementary material for: Linkage between Fitness of Yeast Cells and Adenylate Kinase Catalysis
Source: PLoS One. 2016 Sep 19;11(9):e0163115. doi: 10.1371/journal.pone.0163115 (PMC5028032; doi:10.1371/journal.pone.0163115)
Supplement: S1 Fig — The velocity of ADP production (V with unit M s-1) is scaled by the enzyme concentration (V/[Adk]) to obtain the displayed parameter of the y-axis with the unit s-1. The assays were performed with an AMP concentration held constant at 300 μM at 20°C. Error bars are obtained from the standard deviation resulting from three technical replicates. Displayed are the unique variants analyzed in this study and also Adkeco1.00 (A) Adk1yeast. (B) Adkeco1.00 (C) Adkeco0.47. (D) Adkeco0.20. (E) Adkeco0.12. (F) Adkeco0.06. (G) Adkeco0.007. (DOCX) [file pone.0163115.s001.docx]

*
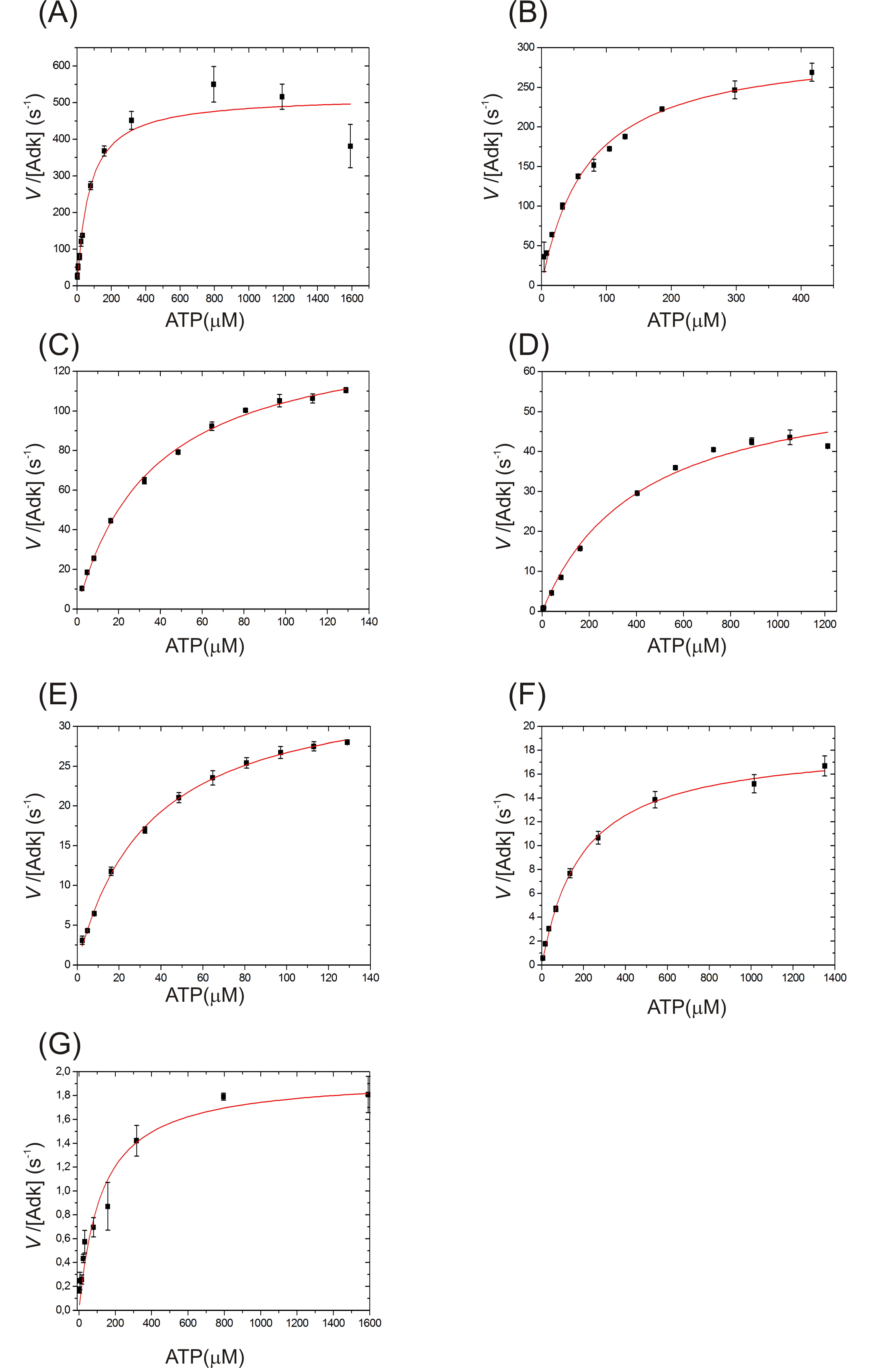
*

**S1 Fig.** Enzyme kinetics with ATP as variable substrate. The velocity of ADP production, *V* with unit M s^-1^, obtained from fitting the experimental data to equation (1) is scaled by the enzyme concentration (*V*/[Adk]) to obtain the displayed parameter of the y-axis with the unit s^-1^. The assays were performed with an AMP concentration held constant at 300 µM at 20 °C. Error bars are obtained from the standard deviation resulting from three technical replicates. Displayed are the Adk variants analyzed in this study. (**A**) Adk1_yeast_. (**B**) $\text{Adk}_{\text{eco}}^{\text{1.00}}$ (**C**) $\text{Adk}_{\text{eco}}^{\text{0.47}}.$(**D**)$\text{Adk}_{\text{eco}}^{\text{0.20}}$. (**E**) $\text{Adk}_{\text{eco}}^{\text{0.12}}$. (**F**)$\text{Adk}_{\text{eco}}^{\text{0.06}}$. (**G**)$\text{Adk}_{\text{eco}}^{\text{0.007}}$
