# Supplementary figures and images for: Linkage between Fitness of Yeast Cells and Adenylate Kinase Catalysis
Source: PLoS One. 2016 Sep 19;11(9):e0163115. doi: 10.1371/journal.pone.0163115 (PMC5028032; doi:10.1371/journal.pone.0163115)

**
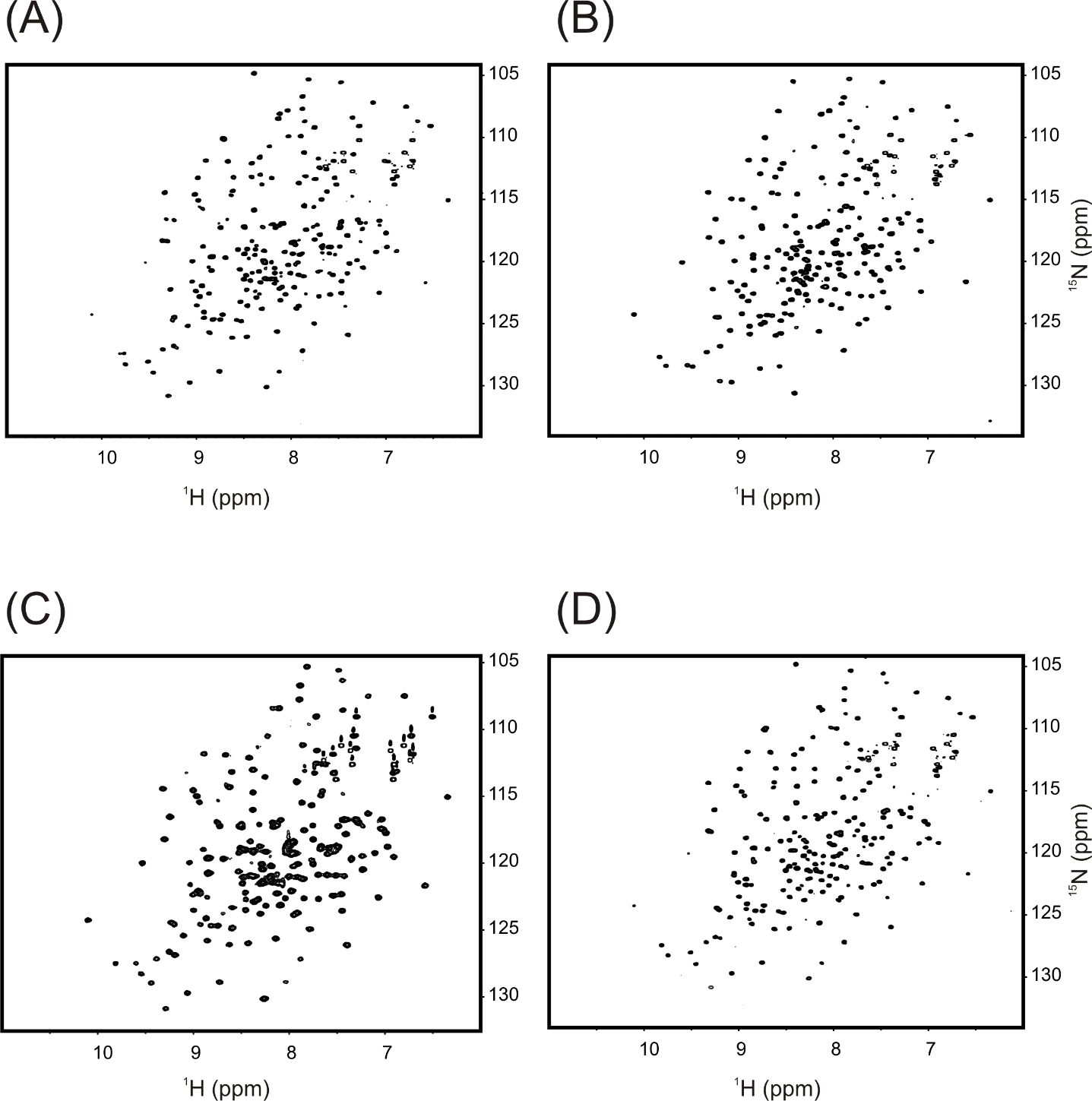
**

**S2 Fig.** 1H-15N HSQC spectra of Adkeco variants. (**A**) T163C. (**B**) G10V. (**C**) G56C. (**D**) R36A.

Supplement: S2 Fig — (A) T163C. (B) G10V. (C) G56C. (D) R36A. (DOCX) [file pone.0163115.s002.docx]

**
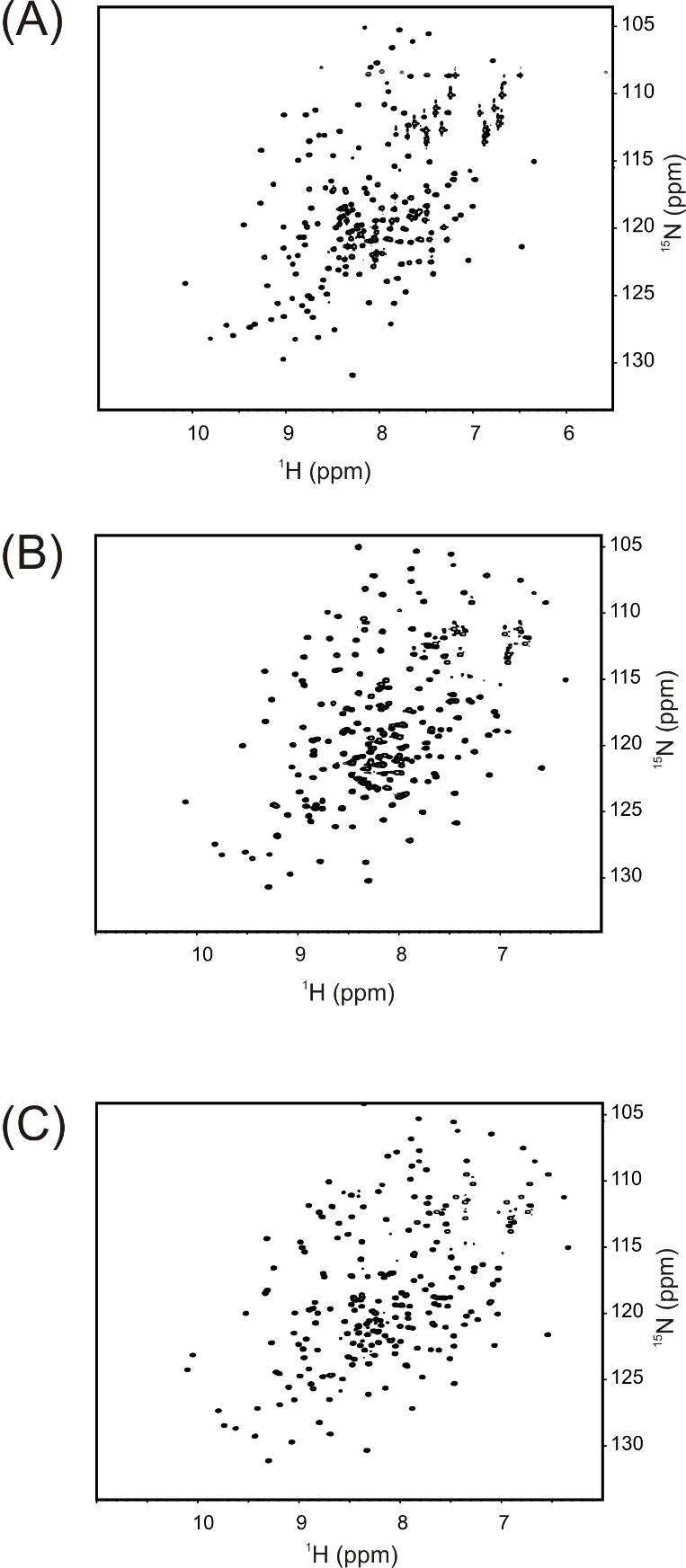
**

**S3 Fig.** 1H-15N HSQC spectra of Adkeco variants. (**A**) I116G. (**B**) R36S+11a.a. (**C**) K13Q.

Supplement: S3 Fig — (A) I116G. (B) R36S+11a.a. (C) K13Q. (DOCX) [file pone.0163115.s003.docx]
