# Supplementary material for: Linkage between Fitness of Yeast Cells and Adenylate Kinase Catalysis
Source: PLoS One. 2016 Sep 19;11(9):e0163115. doi: 10.1371/journal.pone.0163115 (PMC5028032; doi:10.1371/journal.pone.0163115)
Supplement: S1 Table — (DOCX) [file pone.0163115.s004.docx]

| **S1 Table.** Yeast strains used in this study | | |
| --- | --- | --- |
| **Yeast strain** | **Genotype** | **Source** |
| UMY3387 | *MATa/MATα ura3-1/ura3-1 leu2-3,112/leu2-3,12 trp1-1/trp1-1 his3-11,15/his3-11,15 can1-100/can1-100 ade2-1/ade2-1 SSD1-v1/SSD1-v1* | [1] |
| UMY3669 | *MATa/MATα ura3-1/ura3-1 leu2-3,112/leu2-3,12 trp1-1/trp1-1 his3-11,15/his3-11,15 can1-100/can1-100 ade2-1/ade2-1 SSD1-v1/SSD1-v1 ADK1/adk1::kanMX* | This study |
| UMY3974 | *MATa ura3-1 leu2-3,12 trp1-1 his3-11,15 can1-100 ade2-1 SSD1-v1 adk1::kanMX +* pRS316-*ADK1* | This study |

**Supporting reference**

1. Huang B, Lu J, Bystrom AS (2008) A genome-wide screen identifies genes required for formation of the wobble nucleoside 5-methoxycarbonylmethyl-2-thiouridine in Saccharomyces cerevisiae. Rna-a Publication of the Rna Society 14: 2183-2194.
